# Radiographic and clinical assessment of unidirectional porous hydroxyapatite to treat benign bone tumors

**DOI:** 10.1038/s41598-020-78409-9

**Published:** 2020-12-09

**Authors:** Toshiyuki Kunisada, Joe Hasei, Tomohiro Fujiwara, Eiji Nakata, Suguru Yokoo, Koji Demiya, Toshifumi Ozaki

**Affiliations:** 1 Department of Orthopaedic Surgery, Okayama University Graduate School of Medicine, Dentistry, and Pharmaceutical Sciences, 2-5-1, Shikata-cho, Okayama, 700-8558 Japan; 2 Department of Medical Materials for Musculoskeletal Reconstruction, Okayama University Graduate School of Medicine, Dentistry, and Pharmaceutical Sciences, 2-5-1, Shikata-cho, Okayama, 700-8558 Japan

**Keywords:** Orthopaedics, Biomedical materials

## Abstract

Unidirectional porous hydroxyapatite (UDPHAp) was developed as an excellent scaffold with unidirectional pores oriented in the horizontal direction with interpore connections. The purpose of this study was to assess radiographic changes and clinical outcomes and complications following UDPHAp implantation to treat benign bone tumors. We retrospectively analyzed 44 patients treated with intralesional resection and UDPHAp implantation for benign bone tumors between 2010 and 2015. Clinical and radiographic findings were evaluated postoperatively at regular follow-up visits. The mean follow-up was 49 months. Radiographic changes were classified into five stages based on bone formation in the implanted UDPHAp according to Tamai’s classification. All patients showed excellent bone formation inside and around implanted UDPHAp. Absorption of UDPHAp and bone marrow cavity remodeling was identified in 20 patients at a mean of 17 months postoperatively, and was significantly more common in young patients. Preoperative cortical thinning was completely regenerated in 26 of 31 patients on average 10 months after surgery. There were no cases of delayed wound healing, postoperative infection, or allergic reaction related to implanted UDPHAp. UDPHAp is a useful bone-filling substitute for treating benign bone tumor, and the use of this material has a low complication rate.

## Introduction

Benign bone tumors can be treated surgically when they cause severe symptoms or pain or increase the risk of pathological fracture. Imaging with radiography or computed tomography (CT) often shows osteolytic changes with bony erosion or cortical bone thinning due to these tumors. Intralesional resection (curettage) is a standard surgical procedure for this condition. Due to their high rate of local recurrence, aggressive benign bone tumors such as giant cell tumors and chondroblastomas are usually treated with intralesional resection using a high-speed burr with other adjuvant procedures. Tumor resection often results in more severe cortical bone thinning. Horstmann et al.^1^ studied postoperative changes of cortical thinning in the treatment of benign bone tumors, and reported that cortical thickness normalized in 80% of cases one year after tumor resection and filling with bone substitutes. It is important to fill the cavity left after intralesional resection to restore mechanical strength, although it depends on the size of the defect, the anatomical location, and the functional demands and expectations of the patient.

**Figure 1:**
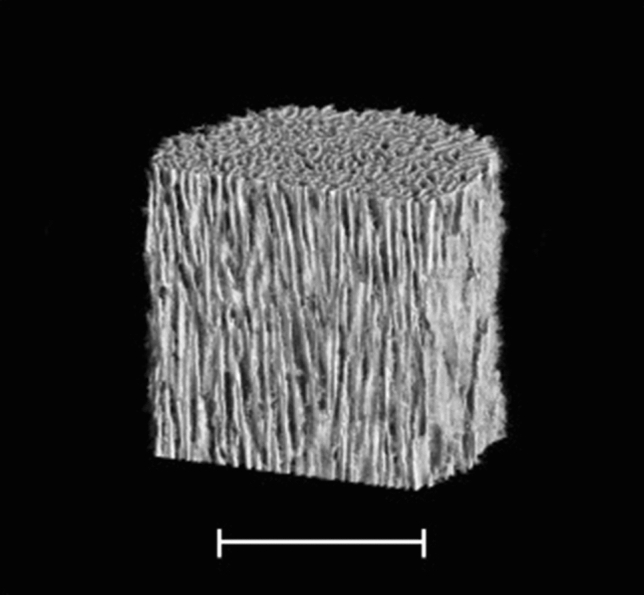
Three-dimensional micro-CT image of UDPHAp (provided by Kuraray Co., Ltd.), showing the unidirectional pores in the vertical direction and some interconnection toward the horizontal direction. Bar; 1000 µm.

Autogenous bone grafting has long been considered the gold standard. However, some complications have been reported, including fracture and infection of the donor site, increased surgery time, greater blood loss, and limited bone supply^2^. The defect after resection may be too large to be filled with autogenous bone alone, and various materials have been used to fill the cavities, including allogenic bone, bone substitute, and polymethylmethacrylate^3^. Hydroxyapatite (HAp) has been widely used as a synthetic graft material following bone tumor resection. As Tamai et al. described, the pores of implanted first-generation HAp rarely filled with newly formed host bone, probably due to the closed structure of HAp with few interpore connections^4^. To overcome this disadvantage, second-generation porous HAp implants with interpore connections of adequate diameter have been developed. Unidirectional porous hydroxyapatite (UDPHAp) consists of unidirectional oval pores oriented toward the horizontal direction that completely penetrate through the material with interpore connections (Fig. 1). The unidirectional porous feature replicates the orientational structure of collagen and HAp of long bone^5^, and increase bone compressive strength in the longitudinal direction with maximum 13.1 MPa compared with the directionless porous structure^6^. Furtehrmore, osteogenic cells are able to penetrate deeply and easily into UDPHAp due to the unidirectional orientation with diameters of 100–300 µm^5,7^. These features facilitate new bone formation and remodeling within UDPHAp^8,9^. UDPHAp has been used to fill bone defects in orthopedic surgeries for high tibial osteotomy and cervical decompression^10,11^. To our knowledge, there has been no English-language report regarding UDPHAp implantation into cavities after benign bone tumor resection. Therefore, we aimed to analyze radiographic and clinical outcomes in patients who underwent UDPHAp implantation after surgical resection of benign bone tumors. We hypothesized that implantation of UDPHAp facilitates bone formation and remodeling in bone cavities after benign bone tumor resection and provides structural and biological benefits in the treatment of cortical thinning due to bone tumors.

## Results

### Radiographic outcomes

**Figure 2:**
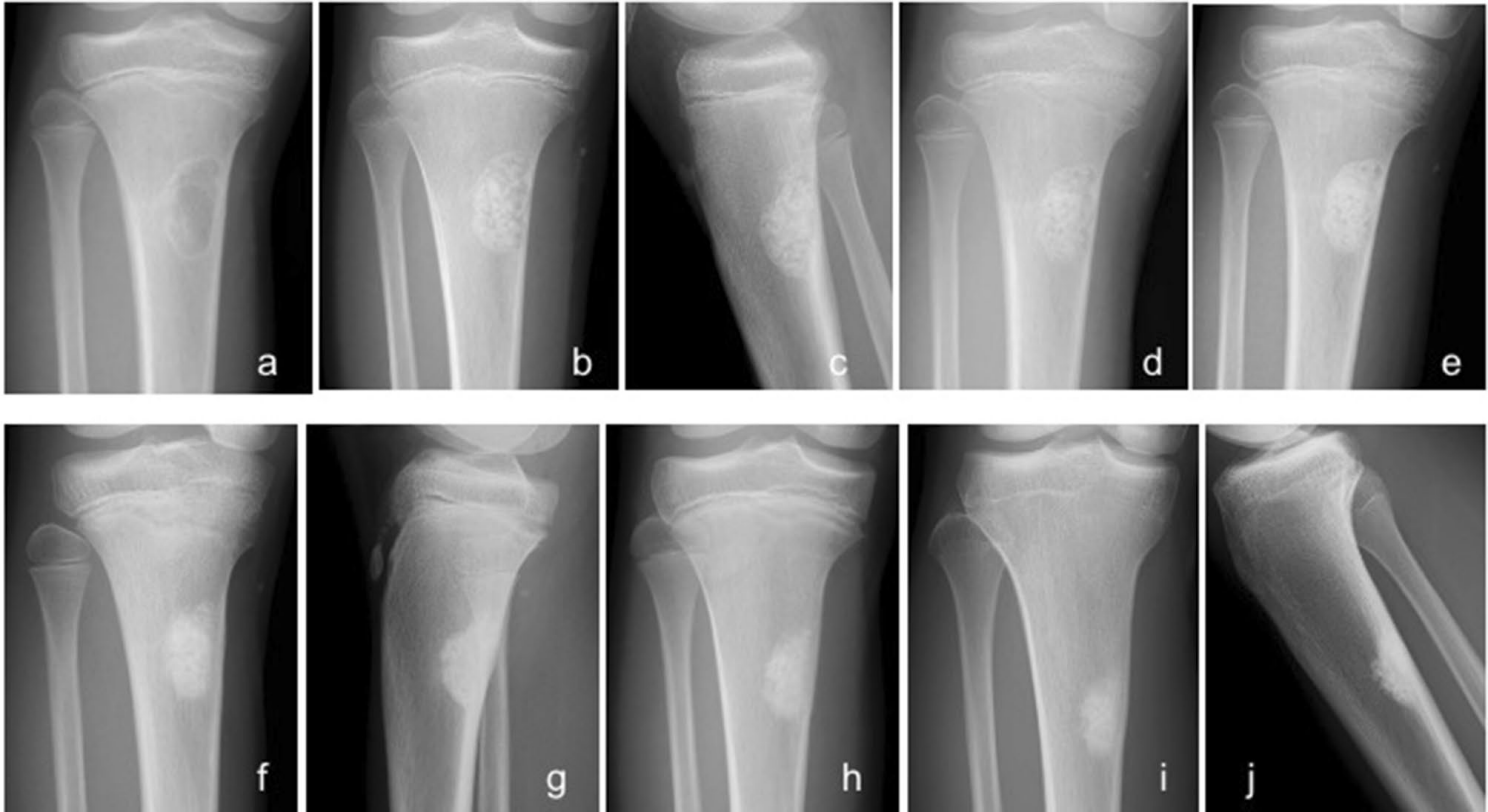
Radiographs of a 9-year-old non-ossifying fibroma of the left tibia treated with granule-type UDPHAp only. (**a**) A lytic change with marginal sclerosis was identified. (**b**) A clear UDPHAp margin was observed one week postoperatively (Stage 0) on anterior–posterior (AP) view and (**c**) lateral view. (**d**) Slight bone formation was noted 1 month postoperatively (Stage 1). (**e**) Moderate bone formation was identified in UDPHAp 3 months postoperatively (Stage 2). (**f**) Diffuse sclerosis was seen with no clear outline of UDPHAp 7 months postoperatively (Stage 3) on AP view and (**g**) lateral view. (**h**) Absorption of UDPHAp and bone marrow remodeling were observed 19 months postoperatively (Stage 4). (**i**) UDPHAp absorption proceeded, and sclerosis was still seen 5 years 3 months postoperatively on AP view and (**j**) lateral view.

**Figure 3:**
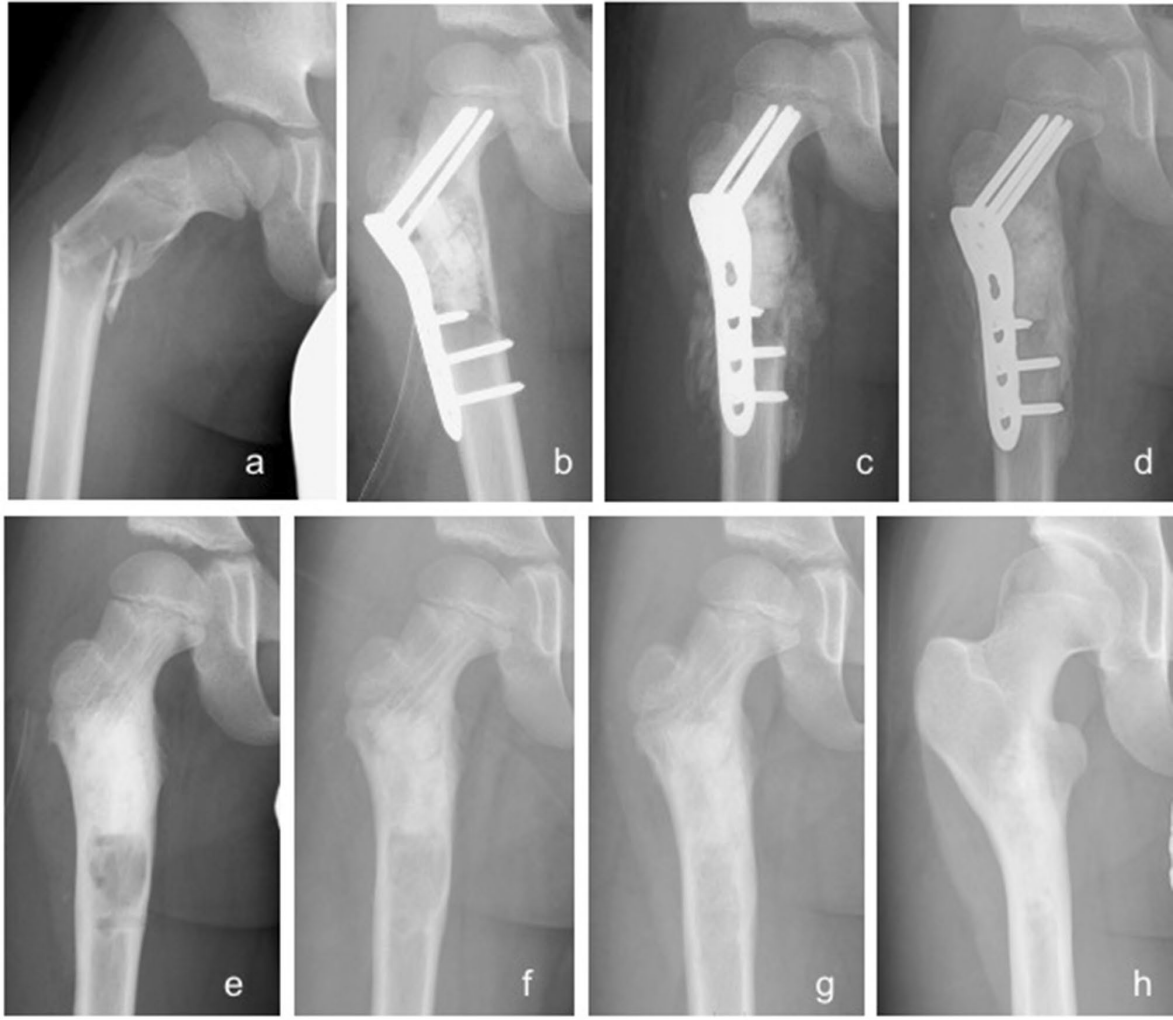
Radiographs of an 8-year-old simple bone cyst of the right proximal femur treated with both block-type and granule-type UDPHAp. (**a**) There was a lytic change and pathological fracture with severe deformity of proximal femur. (**b**) Open reduction and internal fixation with UDPHAp implantation were conducted (Stage 0). (**c**) Moderate bone formation in UDPHAp with a callus around the fracture were observed 2 weeks postoperatively (Stage 2). (**d**) The fracture was healed 2 months postoperatively. (**e**) Internal fixation was removed 11 months postoperatively, and diffuse sclerosis was seen (Stage 3). Local recurrence was suspected and treated with curettage through screw holes at the time of implant removal. (**f**) Absorption of UDPHAp and bone marrow remodeling was noted 13 months after the initial surgery (Stage 4). (**g**) There was no local recurrence 16 months postoperatively. (**h**) UDPHAp absorption proceeded, and slight sclerosis was observed 9 years 6 months postoperatively.

New bone formation was identified inside and around implanted UDPHAp following bone tumor resection and all patients demonstrated at least stage 3 according to Tamai’s staging. Furthermore, 20 patients demonstrated stage 4 with absorption of the material and bone marrow cavity remodeling. The mean postoperative duration to show stage 1 on radiograph was 1.9 months (range 1–5 months); stage 2, 5.8 months (range 2–13 months); stage 3, 12.0 months (range 3–24 months); and stage 4, 17.1 months (range 6–41 months) (Figs. 2 and 3). All nine preoperative pathological fractures were completely healed within 3 months after surgery. The implanted UDPHAp proceeded to stage 4 in six of nine patients with pathological fracture, and stage 3 in three patients.

Table 1 shows the relationship between radiologic assessment at the final follow-up (stage 3 or 4) and clinical factors. Chi-square tests showed that age ≤ 15 years old was a statistically significant indicator for absorption of implanted UDPHAp and bone marrow remodeling (p = 0.04). Gender, type of bone, site in long tubular bone, pathological fracture, volume of implanted UDPHAp, and follow-up period did not show significant associations with stage 4 on the radiograph at the final follow-up.

**Table 1:** Clinical factors influencing radiographic assessment at the final follow-up.

Characteristics	n	Final radiographic assessment		P value
		Stage 3	Stage 4	
Total	44	24	20	
Gender				
Male	30	19	11	0.09
Female	14	5	9	
Age				
≤ 15 years	17	6	11	0.04
> 15 years	27	18	9	
Type of bone				
Long tubular bone	33	17	16	0.48
Non-long tubular bone	11	7	4	
Site in long tubular bone				
Including diaphysis	16	11	5	0.06
Metaphysis or epiphysis only	17	6	11	
Pathological fracture				
Yes	9	3	6	0.15
No	35	21	14	
UDPHAp volume (g)				
≤ 5	31	17	14	0.95
> 5	13	7	6	
Follow-up period (months)				
≤ 36	23	15	8	0.14
> 36	21	9	12	

The mean postoperative durations to stages 2 and 3 were 4.7 and 9.7 months, respectively, for patients with stage 4 at the final follow-up, and 6.6 and 13.9 months for those with stage 3 at the final follow-up, with significant statistical differences between the two groups (p = 0.04 and p = 0.02, Table 2). Diffuse sclerosis due to new bone formation was identified significantly earlier in the stage 4 group than in the stage 3 group.

**Table 2:** Durations (in months) of radiographic stages at the final follow-up.

	Final radiographic assessment		P value
	Stage 3	Stage 4	
Mean duration to stage 1	2.0	1.6	0.52
Mean duration to stage 2	6.6	4.7	0.04
Mean duration to stage 3	13.9	9.7	0.02

**Figure 4:**
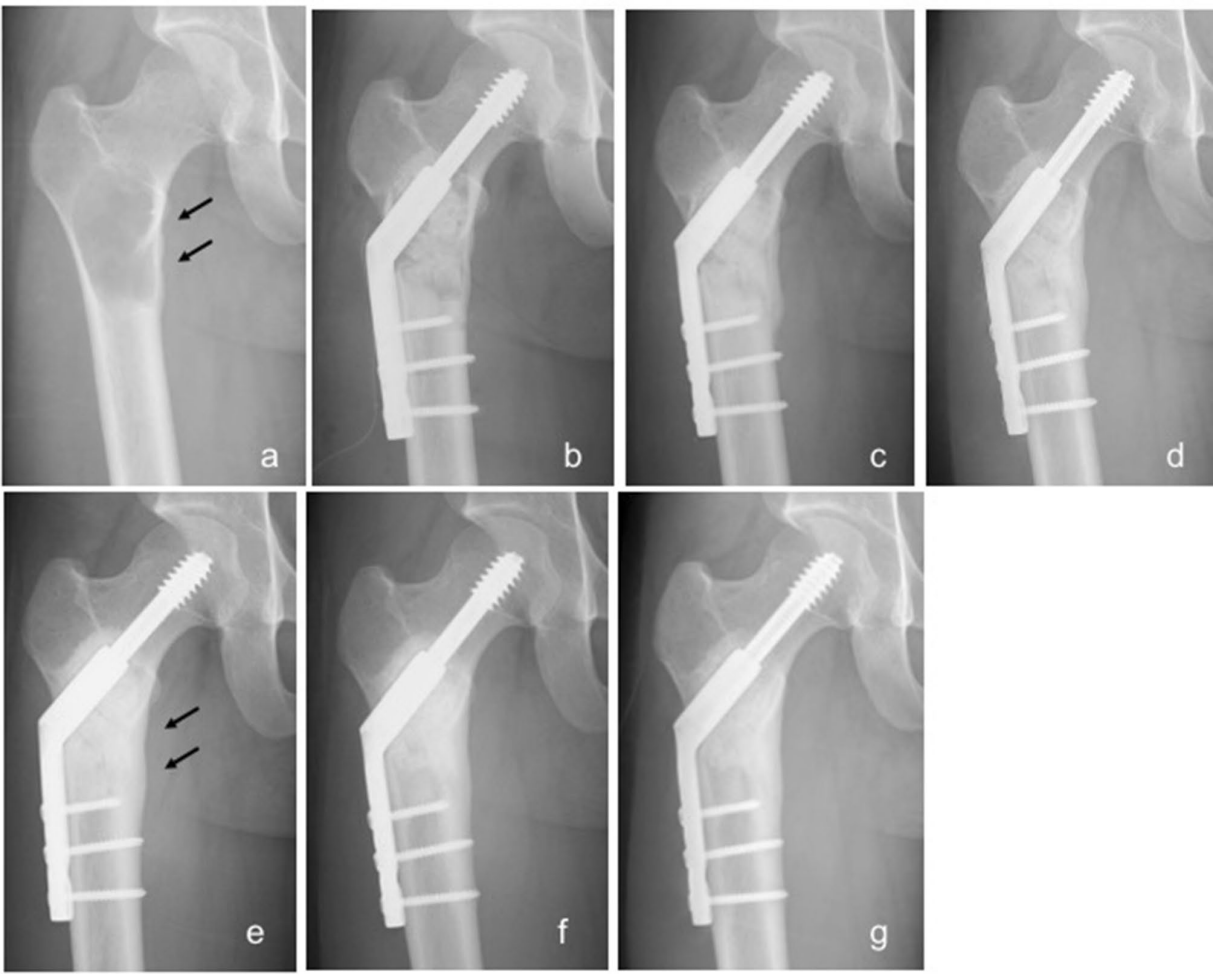
Radiographs of a 23-year-old simple bone cyst of the right proximal femur treated with both block-type and granule-type UDPHAp. (**a**) A lytic change with cortical thinning of the medial bone cortex (arrows) was identified. (**b**) UDPHAp implantation and internal fixation were conducted to prevent fracture. (**c**) Slight bone formation was seen 2 months postoperatively (stage 1). (**d**) Stage 2 radiographic change was confirmed 5 months postoperatively. (**e**) Regeneration of medial cortical thinning (arrows) was seen 10 months postoperatively. (**f**) Stage 3 was noted 19 months postoperatively. (**g**) UDPHAp absorption and bone marrow remodeling were confirmed 3 years postoperatively (Stage 4). The decrease of medial cortical thickness was completely repaired.

Of 33 tumors arising from long tubular bone, 31 demonstrated cortical thinning due to the bone tumor on preoperative radiographs. Regeneration of cortical thinning was observed in 26 patients at an average of 10 months after tumor resection followed by UDPHAp implantation (Fig. 4). Five patients demonstrated only partial regeneration of thinned bone cortex on the final radiograph. Table 3 shows the relationship between regeneration of preoperative cortical thinning due to the tumor and clinical factors, and gender, age, site in long tubular bone, pathological fracture, and volume of implanted UDPHAp did not show significant associations with regeneration of cortical thickness.

**Table 3:** Clinical factors influencing regeneration of preoperative cortical thinning.

Characteristics	n	Regeneration of cortical thickness		P value
		Partial	Complete	
Total	31	5	26	
Gender				
Male	22	4	18	0.63
Female	9	1	8	
Age				
≤ 15 years	9	0	9	0.12
> 15 years	22	5	17	
Site in long tubular bone				
Including diaphysis	15	4	11	0.12
Metaphysis or epiphysis only	16	1	15	
Pathological fracture				
Yes	9	0	9	0.12
No	22	5	17	
UDPHAp volume (g)				
≤ 5	21	5	16	0.09
> 5	10	0	10	

### Clinical outcome

There were no cases of delayed wound healing, postoperative infection, or allergic reaction related to implanted UDPHAp. One SBC patient with a preoperative pathological fracture of the proximal femur fell down the stairs and developed fracture 9 months after implant removal. The patient again underwent internal fixation and showed good bone healing. One patient with ABC in the diaphysis of the humerus showed postoperative radial nerve palsy, that was followed up conservatively at the outpatient clinic and completely recovered 6 months after surgery.

Two patients had local recurrence. One patient with SBC and preoperative fracture was surgically treated with UDPHAp grafting and internal fixation using a plate, and an osteolytic lesion at the distal part was identified 6 months after surgery. The local recurrence of SBC gradually expanded and was treated with curettage alone when the internal fixation was removed (Fig. 3e), resulting in complete healing 5 months after curettage (Fig. 3g). One patient with GCT and preoperative fracture developed local recurrence 52 months after extended intralesional resection and denosumab treatment was initiated. The patient was still treated with denosumab at the final follow-up, and the radiograph showed stable disease of the recurrent tumor.

## Discussion

UDPHAp is a bone filling material with 75% porosity and 99.9% purity that also possesses an interconnected porous structure consisting of unidirectional oval pores toward the horizontal direction (approximately 100–300 µm in the longest diameter) that completely penetrate through the material^9^. The pore size and microstructure of UDPHAp facilitated the invasion of cells and fluids necessary for osteogenesis^8^. Owing to these features, histological analysis showed good bone regeneration and remodeling within UDPHAp^8,9^, and good clinical outcomes of UDPHAp implantation in various bone defects have been also reported^10,12^. We therefore began to use UDPHAp in the treatment of benign bone tumors, which to our knowledge has not been reported. In the present study, UDPHAp implantation resulted in good bone formation in defects with few complications following intralesional resection of benign bone tumors.

Interconnected porous calcium hydroxyapatite ceramic (IP-CHA) was developed as a second-generation HAp with 75% porosity and was expected to be a useful scaffold material for bone tissue engineering^13^. IP-CHA has excellent interpore connections that enable superior osteoconduction by allowing cells and tissues to invade deep into the pores compared with conventional HAp, which has few interconnected pores. UDPHAp can be also a scaffold with continuous communicated pore structure in the axial direction. Tanaka et al.^7^ demonstrated that UDPHAp showed more cells, rhBMP-2, and vessel formation into pores than IP-CHA, indicating that UDPHAp can facilitate early bone formation. In the present study, implanted UDPHAp showed diffuse sclerosis in all patients, and the outlines of granule- and block-type UDPHAp could not be observed on the radiograph (stage 3). Notably, 20 of 44 patients showed good bone marrow cavity remodeling and UDPHAp absorption (stage 4). This change was shown significantly more common in young patients (age ≤ 15). Tamai et al.^4^ reported similar results with IP-CHA implantation after benign bone tumor resection, although they mainly implanted granule-type IP-CHA alone into the cavity. Notably, UDPHAp implantation achieved more stage 3 and 4 radiographic assessments at the final follow-up and shorter durations to stages 3 and 4, indicating that UDPHAp can stimulate early and reliable bone formation, making it an excellent material to fill bony defects.

Histological evaluation revealed that HAp implantation into defects could enhance bone repair processes compared with blood clot only as a control^14^. However, authors^15,16^ argued that bone defects following tumor resection did not routinely require bone filling. In the present study, all preoperative pathological fractures were healed within 3 months after tumor resection followed by UDPHAp implantation, and 84% of cortical thinning around the tumor was regenerated at a mean of 10 months postoperatively. Horstmann et al.^1^ reported similar cortical bone thickness results using a composite ceramic bone graft substitute. There have been few other studies on postoperative radiographic change in cortical thinning due to bone tumors. We believe that filling bone defects with UDPHAp provides structural and biological benefits in the treatment of pathological fractures or cortical thinning due to benign bone tumors.

One patient with SBC fell down the stairs and refractured the bone 9 months after implant removal. We consider that this was an accident and not associated with implanted UDPHAp. The patient underwent internal fixation again, and could perform normal daily activity with stage 4 radiographic assessment at the final follow-up. Some studies reported that postoperative fracture occurred in the early postoperative period after bone grafting with HAp for benign bone tumors^17–19^ and recommended that full weight-bearing should be delayed for at least 3 months for patients with lower extremity tumors^18^. To reinforce bone strength and avoid postoperative fractures, we generally use internal fixation in patients with preoperative pathological fractures or impending fractures due to cortical impairment of tumor extension. There were no fractures in the early postoperative period in this study.

Postoperative radial nerve palsy was observed in one patient with ABC in the diaphysis of the humerus. We exposed the tumor through an anterior approach with medial retraction of the biceps and splitting the brachialis; the radial nerve was not exposed in the surgical field. Radial nerve palsy might be related to retraction of the surrounding soft tissue for surgical exposure, not due to the UDPHAp implantation. The patient completely recovered 6 months after surgery without additional treatment.

The results of this study should be considered in the context of several important limitations. First, there was no comparison group or randomization. We described patients who underwent intralesional resection followed by UDPHAp implantation, and we cannot compare our results with other types of bone filling materials or no filling, because all patients received the same treatment. Owing to the variety of size, location, cortical thickness, and bone tumor histologies, we were unable to provide a control group for comparison. Second, there was no three-dimensional imaging assessment. CT may be able to more precisely show new bone formation and incorporation of implanted UDPHAp, but repeated CT assessment was not appropriate due to the ethical issue of radiation exposure. We believe that plain radiographs can be useful to evaluate such changes. Third, since benign bone tumors generally occur in young patients, most of patients in our series were relatively young with an average age of 24 years and might show better bone formation than elderly patients. Although our results may not be able to lead to the usefulness of UDPHAp implantation in older population, some previous papers have reported its good clinical outcome in the treatment of degenerative diseases such as osteoarthritis^10^ and cervical myelopathy^11^. We believe that UDPHAp can also be a reliable bone graft substitute for elderly patients.

In conclusion, early and excellent bone formation and remodeling was observed on radiographs after bone tumor resection and filling the bone defect with UDPHAp. Cortical thickness was generally increased following tumor resection and UDPHAp implantation. Our results demonstrate that UDPHAp is a useful bone-filling substitute to treat benign bone tumors, and its use has a low complication rate.

### Patients and methods

We retrospectively analyzed 44 patients treated with intralesional resection (curettage) and implantation of UDPHAp (REGENOS, Kuraray Co., Ltd., Tokyo, Japan) for benign bone tumors between 2010 and 2015. There were 30 males and 14 females with an average age of 24 years (range 8–72 years). The mean follow-up was 49 months (range 12–116 months). The histologies included 16 simple bone cyst (SBC), eight enchondroma (EC), six giant cell tumor of bone (GCT), six fibrous dysplasia (FD), four chondroblastoma (CB), three non-ossifying fibroma (NOF), and one aneurysmal bone cyst (ABC). Twenty-nine tumors were located in lower extremities (17 femur, 6 calcaneus, 2 tibia and 4 others), 12 in upper extremities (five humerus, four metacarpal, two phalanx, and one radius), and 3 in the pelvis. Patients who were surgically treated for local recurrence were excluded.

We conducted intralesional resection by manual curettage alone for less aggressive benign bone tumors such as SBC, FD, EC, or NOF. Intralesional resection with a high-speed burr was performed for aggressive benign bone tumors such as GCT, ABC, or CB. An argon beam coagulator, soaking with alcohol, and/or irrigation with distilled water were used as adjuvant therapies for GCT.

Adequate amounts of both block-type and granule-type UDPHAp were implanted into bone defects after intralesional resection of the tumor in 24 patients, and only granule-type UDPHAp was implanted in 20 patients. Bone defects < 3 cm in longitudinal diameter were generally filled with granule-type UDPHAp alone. Block-type UDPHAp was grafted in the cavity with its pores parallel to the longitudinal direction of bone, as shown in previous reports^8,10^. The average volume of implanted UDPHAp was 7.5 g (range 1–46 g). Autogenous bone was simultaneously grafted into subchondral bone defects adjacent to the joint in three patients. Internal fixation with plates and screws was used in ten patients to repair the pathological fracture that presented at the time of diagnosis or to prevent postoperative fracture and facilitate rehabilitation.

Regular postoperative radiographs were taken at the outpatient clinic. Two orthopedic surgeons in our department (JH and TF), who were blinded to the clinical conditions assessed radiographic changes in implanted UDPHAp according to Tamai’s staging^4^ It is divided into five stages based on bone formation in the implanted UDPHAp: stage 0, no any bone formation in the UDPHAp; stage 1, slight bone formation in the UDPHAp with radiolucency superior to that of neighboring cortical bone; stage 2, moderate bone formation in the UDPHAp with radiolucency less than or equal to that of neighboring cortical bone; stage 3, consolidation stage characterized by diffuse extensive sclerosis without visible outline of the UDPHAp granules; and stage 4, absorption stage (absorption of the UDPHAp or remodeling of the bone marrow cavity). Clinical complications were abstracted by chart review and performed by the treating surgeon (TK).

Chi-square tests were used to assess differences in proportions between the two groups. Univariate analyses were performed using Mann–Whitney U tests for non-parametric data. For all tests, p < 0.05 was considered statistically significant. The software program PASW statistics (version 18, SPSS Inc., Chicago, IL) was used for all analyses.

The study was approved by the Institutional Review Board of Okayama University (Rin#1262). All methods were performed in accordance with the relevant guidelines and regulations. Written informed consent to participate in this study was obtained from the patients or their parents.

## Data Availability

The datasets generated during and/or analyzed during the current study are not publicly available due to individual patient privacy laws which may be compromised but are available from the corresponding author on reasonable request.
